# Synergistic Effect Between Online Broadcast Media and Interactive Media on Purchase Intention

**DOI:** 10.3389/fpsyg.2021.781743

**Published:** 2021-12-23

**Authors:** Yin Gao, Lin Zhao

**Affiliations:** ^1^Department of Marketing, College of Business Administration, Shanxi University of Finance and Economics, Taiyuan, China; ^2^Research Secretary, School of Public Administration, Shanxi University of Finance and Economics, Taiyuan, China

**Keywords:** media synergy, online broadcast media, online interactive media, product memory, online purchase intention

## Abstract

It is becoming increasingly important for marketers to use online broadcast media and interactive media simultaneously. In this paper, three studies were conducted to explain the mechanism inherent in a synergistic effect. We ran Study 1 to test the synergistic effect of online broadcast media and interactive media on purchase behavior. We conducted Study 2 (synergistic vs. non-synergistic groups) to test the differences between the groups. Study 2 was a 2 (interference vs. no interference terms) × 2 (synergistic vs. non-synergistic groups) experiment to test whether the interaction between online broadcast media and interactive media is a necessary condition for the synergistic effect. Finally, Study 3 was conducted to test the difference in the level of information processing between online broadcast media and interactive media as a necessary condition for the synergistic effect. We designed a 2 (questioning vs. not questioning) × 2 (synergistic vs. non-synergistic groups) experiment to examine the difference between the groups. The results show that online broadcast media and interactive media have a synergistic effect on consumer purchase intention and memory. Study 1 shows that participants in the synergistic group had a higher purchase intention than participants in the non-synergistic group. Study 2 revealed a significant cognition interaction effect on product memory between online broadcast media and interactive media. Finally, in Study 3, the difference in the information processing level between broadcast media and interactive media had a significant synergistic effect on product memory.

## Introduction

In the last 20 years, online marketing has rapidly become a favored method for conveying corporate advertising, promotions, public relations, and other communications (Stephen and Galak, 2012). The introduction of deploying online broadcast media and interactive media simultaneously has presented both opportunities and challenges for marketing managers. On one hand, it is important to select the right combination of online media as certain media may be incompatible and may result in “1 + 1 < 2” (Naik and Raman, 2003). On the other hand, based on numerous direct experiments, marketing managers have found that including interactive media is essential to getting the results that exceed expectations when devoting a large budget to broadcast advertising (Culnan et al., 2010; Dong et al., 2018). For example, Ford obtained good results when promoting the new Ford Focus model through Facebook, Twitter, and other interactive media (Levin, 2011). Only a few studies have focused on online multimedia marketing issues (Schultz et al., 2012). However, we still know little about the online multimedia synergistic effect. It is important for marketers to understand how online broadcast media interact with interactive media and how the interaction affects consumer variables.

Synergistic effect is the basis of synergy research. Previous studies have found that media with different sensory patterns, origins, and information load can produce a synergistic effect (Maheswaran and Chaiken, 1991; Dijkstra et al., 2005; Chatterjee, 2012). Differences in the form of media can lead to a synergistic effect because of the sensory model, the origin of information, or the different information load. Therefore, based on the consideration of the form to determine content, we hypothesize that a synergistic effect will occur between interactive media and online broadcast media as these two types of media differs greatly in form from the original.

This study analyzes the synergistic effect between online broadcast media and interactive media and shows that media collaboration has an impact on purchase intention through product memory.

## Theoretical Background

### Online Media Synergistic Effect and Purchase Intention

Media synergy refers to multimedia cohesions that exceed the sum of the influence of use alone (Naik and Raman, 2003; Naik and Peters, 2009). In this research, we define the multimedia synergistic effect as the media cohesion that exceeds the sum of the effect of use alone, and synergy as the combination of multimedia.

Research on the influence of media synergy on consumer behavior has focused primarily on three avenues of inquiry. First, research on traditional offline multimedia has found that synergy has a positive effect on purchase intention. For example, the combination of TV and broadcast advertising can increase audiences’ purchase intention (Edell and Keller, 1989). Synergy between TV, print advertising, and other print media can also improve consumers’ brand awareness and purchase intention (Dijkstra et al., 2005).

Second, research on the synergistic effect between traditional offline media and online media on purchase intention has yielded different conclusions. In Chang and Thorson (2004) research, the coordination of TV and web advertising led to higher perceived information trust and more aggressive product ideas for the consumer. However, other studies point out that the combination of TV, print media, and online banners has no effect on purchase intention (Dijkstra et al., 2005).

Third, research on the role of online multimedia synergy on purchase intention is limited. Abraham (2008) found that online display advertising and online search advertising can increase online and offline sales. Other studies have found that acquired social media (such as online communities) have a role in promoting acquired traditional media (Stephen and Galak, 2012).

The research hypothesis is stated as follows:

***H1***: The combination of online broadcast media and interactive media has a synergistic effect on purchase intention.

### Different Media Produce Synergy Through Interaction

Integrated marketing theory points out that there is a complex interaction between the marketing communication tools (Smith et al., 2006). However, we do not know what this “complex interaction” is. Cognitive information processing theory provides a theoretical explanation for the sequential stimulation of different media synergies. After the first advertisement stimulation, the consumer forms memory traces. After the second stimulation, three kinds of cognitive processing activities occur. First, the memory traces of the first advertisement stimulation are retrieved and the second advertisement information provides an “introduction” and a “bait” for memory retrieval. Second, the encoding of current advertisement stimulation information includes two cognitive activities, namely, deepening comprehension and refinement, and thus forms the second memory structure. Third, a new memory structure is formed by way of schematic representation of the first memory traces and the second memory structure (Edell and Keller, 1989). In this information processing, interaction determines the recoverability of the memory traces, thus affecting the processing of the follow-up stimulus. Therefore, we assume Hypothesis H2a as follows:

***H2a***: Online broadcast media and interactive media can only produce a synergistic effect on product memory through an interaction.

### Different Media Produce Synergy Through Form Differences

The collaboration of different media creates higher brand awareness, higher purchase intention, and other marketing results, leading to the effect that overall surplus part. The current research literature focuses on the following three aspects of media form differences.

First, different media produce synergy through different perceptual modes. The greater the number of different sensory modalities, the better the communication and learning effects (Jacoby et al., 1983; Kisielius and Sternthal, 1984). For example, video media and auto media, video media and print media, and video media and web display all can produce synergy, thus resulting in higher advertising memory (Edell and Keller, 1989; Dijkstra et al., 2005; Voorveld et al., 2011). Consumers can even produce higher brand ratings and better advertising memory using the two sensory cues—smells and images (Lwin and Morrin, 2012). In short, media with different sensory modalities provide more coding channels and form multi-tiered cognitive structures (Becker-Olsen, 2003).

Second, different media create synergy through different information loads. Different media forms, such as paper media and email, carry different amounts of information. Different information loads can lead to different levels of mental activation and produce different product evaluations (Keller and Staelin, 1987). Previous research has focused on the collaboration of three kinds of media, namely, network banner, paper media, and email. Although these three media have the same sensory model, their combination can produce higher brand recall and brand attitude due to their different information loads (Chatterjee, 2012).

Third, different media produce synergy through different information sources (media attribution sources). Consumers process information and react differently to different media sources. For example, consumers take different cognitive approaches to media launched by businesses and third parties. Differences in the source of media attribution lead to cognitive differences (Cameron, 1994). Audiences allocate more cognitive ability to understand information, and produce stronger product attitude and new integration evaluation when media with different information sources release coordinated stimulation (Maheswaran and Chaiken, 1991). For example, the two different media sources, advertising and publicity, enhance information inference and purchase intention when deployed together (Wang and Nelson, 2006).

In the abovementioned studies, different media produce synergy because of their form differences, such as different sensory modalities, different information loads, and different information sources. The memory structure stimulated by different media forms is larger than the one produced by an individual media form. That is, the media form difference produces a synergistic effect. Based on these studies, we hypothesize that there is a form difference between online broadcast media and online interactive media that leads to synergy because audiences will produce different levels of information processing.

### Different Information Processing Levels and Product Memory

Online broadcast media, which include the enterprise’s website, network banners, network text, etc., have a remarkable unidirectional characteristic of information communication (Andzulis and Panagopoulos, 2012). After receiving this kind of media stimulation, consumers often choose to ignore information and have no obvious consciousness or thought to participate in information processing. Online interactive media, which include microblogs, network communities, and forums, have a bidirectional characteristic of information communication, problem-solving, and other activities (Haythorthwaite, 2005; Kim and Ko, 2012). The difference between online broadcast media and online interactive media is essential as to whether consumers have significant consciousness and thought to participate in the process of media information processing.

In the process of forming information, the individual has a higher level of information processing under conscious participation compared to unconscious participation. When an individual was aware they are processing information, the brain performs a higher level of information processing (Hulstijn, 1992). Different levels of information processing form different memory structures. The higher the processing level, the more complex and abstract the semantic analysis. The latter is conducive to information retention in long-term memory (Kapur et al., 1994). Therefore, we believe that online broadcast media lead to a low level of consumer information processing, while online interactive media lead to high levels of information processing. The form difference (interactive vs. not interactive) leads to the difference in the information processing level, and a different information processing level can form a strengthened cognitive structure.

Through the difference of information processing level, the collaborative stimulation of online broadcast media and interactive media can push consumers to form a strengthened product memory that exceeds the product memory achieved by either online broadcast media or interactive media individually. In this study, we employed product recall and product identification as the two dimensions of product memory. That is, there is synergy between online broadcast media and interactive media. We assume the following:

***H2b:*** Online broadcast media and online interactive media can only produce a synergistic effect on product memory through different information processing levels.

clearly describe the relationship between variables, a conceptual model is presented in Supplementary Figure A1.

Next, we present the three studies designed to test these hypotheses. In Study 1, we provide evidence for the synergistic effect of online broadcast media and online interactive media on consumer purchase intention. We propose the two conditions (interaction and different processing levels of stimuli) of the synergistic effect between online broadcast media and interactive media. We provide evidence of the theoretical mechanism for the online multimedia synergistic effect in Studies 2 and 3. In Study 2, the first condition interaction is verified by illustrating that the interaction between online broadcast media and interactive media creates a synergistic effect. In Study 3, we demonstrate the second condition that different processing levels of stimuli (online broadcast media and interactive media) can produce a synergistic effect. We close with a discussion of the implications of these findings.

## Study 1

In Study 1, the objective was to provide evidence for H1 by validating the existence of the media synergistic effect.

### Method, Stimuli, and Procedure

One hundred and twenty-eight undergraduate students from Huazhong University of Science and Technology participated in this study for course credit. In this study, a 2 (synergistic and non-synergistic) between-group design was employed. We randomly assigned participants to four groups: Broadcast-Interactive (B-I), Broadcast-Broadcast (B-B), Interactive-Broadcast (I-B), and Interactive-Interactive (I-I), so that the participants of each group were exposed to two media stimulations. We constructed the synergistic group with B-I and I-B and the non-synergistic group with B-B and I-I. We manipulated online media (broadcast media and interactive media) by two websites displaying advertisements (virtual product network home page and virtual product network forum) that a pretest showed, which were significantly different in product attitude (1 = “strongly disagree” and 7 = “strongly agree”; *M*_*homepage*_ = 2.15, *M*_*forum*_ = 3.17; *t* = 7.599, *p* = 0.010). Within the groups, participants were randomly assigned to one of the two online media stimulus conditions. In the online broadcast media stimulus condition, subjects were exposed to an online advertisement embedded in the virtual product network home page. In the online interactive media stimulus condition, subjects were exposed to an advertisement embedded in the virtual product network forum.

For this study and the following studies, we created advertisements using images and advertisement propositions from Baidu. For Study 1, we chose six advertisement images of a mobile phone brand as an experimental material. We used these images to design a virtual phone brand labeled “Dis-80.” Then, to reduce the impact of former brand knowledge and experience, we change the logo of the pictures. Some product advertisement propositions were written by other 28 students participating in the production of experimental product advertisements. All the product propositions were evaluated by the group members, including 1 title and 16 product advertisement propositions. The participants could conduct a series of interactive behaviors, such as inputting comments and answering questions. Finally, advertising portfolios were produced. We combined the two types of pages (virtual mobile phone home page and virtual mobile phone network forum) in pairs through the website linkage and created the four advertising portfolios that represented B-I, I-B, B-B, and I-I, respectively. We set a fixed browsing time for each with an automatic linkage. Taking the advertising portfolio B-I as an example, the browsing time for each web page’s advertisement was 120 s. After a 120-s display of the virtual mobile home page, the virtual mobile phone network forum was opened automatically through a web linkage. The advertising portfolio closed automatically after 120 s of continuous display.

As a cover story, fifty-six subjects were told that they would be pretesting stimuli for a business study on media advertising in a school–enterprise cooperation project and that they could earn school credit through the test. Before beginning, all external networks were removed and subjects were asked general information questions, including gender, age, height, weight, general online media usage habits, and a current product attitude measured on a seven-point scale anchored by 1 (“strongly disagree”) and 7 (“strongly agree”). Next, participants were told that they would be evaluating a mobile phone advertisement. The advertising portfolio on the computer were then shown to participants, and participants were asked to state their purchase intention for Dis-80 on an eleven-point scale anchored by 1 (“1% likelihood of making a purchase”) and 11 (“99% likelihood of making a purchase”). Finally, we included a manipulation check for online media and responded to participants’ questions about the study.

### Results and Discussion

#### Online Media Manipulation

To test the effectiveness of two kinds of media manipulation, a one-way ANCOVA with online media type as a between-subject factor and product attitude as a dependent variable revealed the expected significant effect of online media type such that participants perceived more positive product attitude in the online mobile phone forum (*M*_*forum*_ = 3.17) than in the online mobile phone home page (*M*_*homepage*_ = 2.15; *t* = 7.599, *p* = 0.010).

#### Purchase Intention Analysis

First, we compared synergistic and non-synergistic groups. Using purchase intention as a dependent variable, we conducted a one-way ANCOVA with synergy type (synergy and non-synergy) as a between-group factor. The results revealed a significant effect for synergy type (*M*_*synergy*_ = 2.270, *M*_*non–synergy*_ = 1.400; *t* = 8.659, *p* = 0.004), indicating that the synergy between the Dis-80 advertisement in the home page and the Dis-80 advertisement in the mobile phone forum potentially create a higher purchase intention than the non-synergy case (see Table 1).

**TABLE 1:** Comparison of synergistic stimulation and non-synergistic stimulation.

Variable	Media exposure		*t*	Significance
	Non-synergy (B-B and I-I)	Synergy (B-I and I-B)		
Purchase Intention	1.400	2.270	8.659	0.004

Next, using purchase intention as a dependent variable, we conducted a one-way ANCOVA with synergy type (synergy and separate stimulation) as the between-group factor. Specifically, in accordance with H1, participants exposed to synergy media were more willing to buy the Dis-80 phone (*M*_*synerg*_*_*y*_* = 2.487; *t* = 8.024, *p* = 0.007) than participants exposed to separate stimulation (*M*_*separate B*–B_ = 1.278). Similarly, participants exposed to synergy media were more willing to buy the Dis-80 phone (*M*_*synergy*_ = 2.487; *t* = 5.260, *p* = 0.026) than participants exposed to separate stimulation (*M*_*separate*__*I–I*_ = 1.529) (see Table 2).

**TABLE 2:** Comparison of separate stimulation and synergistic stimulation.

Variable	Media exposure			*t*	Significance
	Separate stimulation (B-B)	Separate stimulation (I-I)	Synergy (B-I and I-B)		
Purchase Intention	1.278	_	2.487	8.024	0.007
	_	1.529	2.487	5.260	0.026

These results support H1. Study 1 verified the synergistic effect between online broadcast media and online interactive media at the behavioral willingness level (purchase intention). Because of differences in media type, audiences are more likely to recognize a product brand. As a result, audiences produce higher product identification and higher purchase intentions for targeted products. This conclusion is consistent with other scholars’ findings that the combination of TV advertising and broadcast advertising can increase willingness to buy (Edell and Keller, 1989). Other studies have focused on other variables in the consumer’s behavioral dimension. For example, Jagpal (1981) found that the combination of a commercial bank’s radio and newspaper advertising could increase media consumption. Similarly, Dijkstra et al. (2005) found that TV media and online media could enhance sales of products, store visits, advertising clicks, etc.

However, a small number of studies reached different conclusions in comparison to our study. For example, one study found that there was no synergistic effect for the combination of TV media, print media, and online banner, and that the combination had nothing to do with consumers’ purchase intention (Havlena et al., 2007). Even so, based on previous studies of the effects of synergy on certain psychological variables (Chang and Thorson, 2004; Dong et al., 2018), this study argues that media synergy plays a role in willingness behavior through certain psychological variables.

## Study 2

We believed that the interaction of the two different stimulations (broadcast and interactive) can only produce synergistic effects. A synergistic effect would occur from the two media stimulations in the case of interaction, and *vice versa*. In Study 2, we explored the effects of “interaction” on product memory to verify hypothesis H2a.

### Pretest

We manipulated the interaction between the two media stimulations by adding an interference item in the middle of the media stimulations. For the interference condition, a web page with nonsense text was inserted into the middle of the virtual online product home page and the network forum. For the non-interference condition, we used a blank web page without text. Seventy-eight undergraduate students completed the pretest in this study.

To examine the effectiveness of the interaction between the two stimuli using product memory as a dependent variable, we conducted a one-way ANCOVA with the interference type (interference and non-interference) as the between-group factor. The results show that participants exposed to the blank page had a higher level of recall of product information (*M*_*non–interference*_ = 5.050; *t* = 23.614, *p* = 0.000) than participants exposed to the web page with nonsense text (*M*_*interference*_ = 3.211). Likewise, the treatments resulted in significant differences in product identification (*M*_*interference*_ = 9.790, *M*_*non–interference*_ = 11.075; *t* = 9.520, *p* = 0.003). The results indicate that the web page with nonsense text interferes with memory retrieval, hindering the formation of a memory structure. In contrast, the blank page did not produce any interference effect, resulting in higher product memory.

### Method, Stimuli, and Procedure

One hundred and eighty-eight undergraduate students participated in this study for course credit. The study employed a 2 (interference group and non-interference group) × 2 (synergistic group and non-synergistic group) between-group design. In this study, we made *d* to represent a web page with nonsense text, and *b* to represent a blank web page. Finally, we randomly assigned participants to eight groups (B-d-I, I-d-B, I-d-I, B-d-B; B-b-I, I-b-B, I-b-I, and B-b-B) each group being exposed to three media stimulations. For the interference condition, we constructed the synergistic group with B-d-I and I-d-B and the non-synergistic group with I-d-I and B-d-B. For the non-interference condition, we constructed the synergistic group with B-b-I and I-b-B and the non-synergistic group with I-b-I and B-b-B.

The experimental stimuli were eight advertising portfolios similar to the ones in Study 1. We similarly combined the four types of pages (a virtual mobile phone home page, a virtual mobile phone network forum, a blank web page, and a web page with nonsense text) in pairs through the web site linkage.

Participants were told that they would be evaluating the use of advertisements that a mobile phone company was considering for a new brand. The advertising portfolio on the computer were then shown to participants, and participants were asked to fill out a product recall scale (the number of recalled words mentioned in the previous advertisement) and a product identification scale (the number of correct true or false statements).

### Results and Discussion

#### Product Memory Analysis

In the non-interference condition, we conducted a one-way ANCOVA with synergy type (non-synergy and synergy) as a between-group factor and product memory as a dependent variable. In Study 2, product recall and product recognition were employed as the two dimensions of product memory. The results (see Table 3) reveal that participants exposed to the synergistic stimulation produced higher levels of product recall (*M*_*synergy*_ = 6.708; *t* = 23.475, *p* = 0.000) than participants exposed to the non-synergistic stimulation (*M*_*non–synergy*_ = 5.050), and produced higher levels of product identification (*M*_*synergy*_ = 12.042; *t* = 11.476, *p* = 0.001) than participants exposed to the non-synergistic stimulation (*M*_*non–synergy*_ = 10.700).

**TABLE 3:** Comparison of non-synergistic stimulation and synergistic stimulation in the non-interference condition.

Variable	Media exposure		*t*	Significance
	Non-synergy (B-b-B and I-b-I)	Synergy (B-b-I and I-b-B)		
Product Recall	5.050	6.708	23.475	0.000
Product Identification	10.700	12.042	11.476	0.001

In the interference condition, we conducted a one-way ANCOVA with the synergy type (non-synergy and synergy) as a between-group factor and product memory as a dependent variable. The results (see Table 4) reveal that product recall did not differ significantly between the non-synergistic group (*M*_*non–synergy*_ = 3.211) and the synergistic group (*M*_*synergy*_ = 3.813); product identification yielded similar results (*M*_*synergy*_ = 9.813, *M*_*non–synergy*_ = 9.790; *t* = 0.03, *p* = 0.957).

**TABLE 4:** Comparison of non-synergistic stimulation and synergistic stimulation in the interference condition.

Variable	Media exposure		*t*	Significance
	Non-synergy (B-b-B and I-b-I)	Synergy (B-b-I and I-b-B)		
Product Recall	3.211	3.813	3.075	0.084
Product Identification	9.790	9.813	0.003	0.957

In this study, we verified that the labeling of two media stimuli with “interaction” is a necessary condition to generate a synergistic effect. In the non-interference group, the inserted blank page did not interfere with the memory structure of the tested product. After the second stimulus, the second stimulus information is integrated with the original memory. Thus, the synergistic stimulation produced a higher product memory than the non-synergistic stimulation. In the interference group, the inserted nonsense text interfered with the participants’ memory of the tested mobile phone as participants could not recall the mobile phone brand. In contrast, participants, instead of remembering the phone brand itself, were more likely to remember the fact there was nonsense text in the home page or the mobile phone forum. This conclusion is consistent with the findings of Harkins and Petty (1981), in which multi-source information can affect the persuasiveness of information and destroy it by obstructing the thought-generating process. The irrelevant material interferes with the retrieval process of the semantic classification table in memory, which leads to an inability to proceed normally (Marsh et al., 2014). Thus, H2a is validated.

## Study 3

We believed that only two different advertising stimulations (broadcast and interactive), that is, media with different information processing levels, could produce synergistic effects. Therefore, Study 3 explored the impacts of two media with different levels of information processing on product memory to verify H2b.

### Pretest

We manipulated the different levels of information processing between the two media stimulations by adding a series of questions to the web pages. For the questioning condition, participants were asked to complete the pop-up questions *via* a link button in the product web forum. For the non-questioning condition, no questions would pop up automatically in the product web forum, even if participants clicked the link button. Forty-two undergraduate students participated in the pretest.

To examine the difference in the effectiveness of information processing between the two media stimulations, we conducted a one-way ANCOVA with questioning type (questioning and non-questioning) as the between-group factor and product memory as a dependent variable. The results show that participants exposed to B-I with automatic pop-up questions had a higher level of recall of product information (*M*_*questioning*_ = 5.050; *t* = 8.719, *p* = 0.005) than participants exposed to B-I without questions (*M*_*non–questioning*_ = 3.211). Likewise, participants had significant differences in product identification (*M*_*questioning*_ = 10.458, *M*_*non–questioning*_ = 9.375; *t* = 3.406, *p* = 0.073). The results indicate that the two types of web forum (questioning and non-questioning) are different in terms of information processing. Because of this, a higher product memory was present in the questioning condition than in the non-questioning condition.

### Method, Stimuli, and Procedure

One hundred and eighty-eight undergraduate students participated in this study for course credit. The study employed a 2 (questioning group and non-questioning group) × 2 (synergistic group and non-synergistic group) between-group design. We randomly assigned participants to eight groups, each group exposed to two media stimulations. For the questioning condition, we constructed the synergistic group with B-I and I-B and the non-synergistic group with I-I and B-B. For the non-questioning condition, we constructed similar groups. Other experimental operations were the same like in Study 1.

The experimental stimuli were eight laptop advertising portfolios. We similarly combined the three types of pages (virtual laptop home page, virtual laptop network forum with pop-up questions, and virtual laptop network forum without automatic pop-up questions) in pairs through the web site linkage, and created eight advertising portfolios.

Participants were told that they would be evaluating the use of advertisements that a laptop company was considering for its new brand. The laptop advertising portfolio on the computer were then shown to participants, and they were asked to fill out a product recall scale (the number of recalled words mentioned in a previous advertisement) and a product identification scale (the number of correct true or false statements).

### Results and Discussion

#### Product Memory Analysis

In the questioning condition, we conducted a one-way ANCOVA with synergy type (non-synergy and synergy) as a between-group factor and product memory as a dependent variable. In Study 3, product recall and product recognition were employed as the two dimensions of product memory. The results (see Table 5) reveal that participants exposed to the synergistic stimulation of laptop advertisement produced higher levels of product recall (*M*_*synergy*_ = 5.833; *t* = 7.378, *p* = 0.010) than participants exposed to the non-synergistic stimulation (*M*_*non–synergy*_ = 4.550), and higher levels of product identification (*M*_*synergy*_ = 10.458; *t* = 4.527, *p* = 0.039) than participants exposed to the non-synergistic stimulation (*M*_*non–synergy*_ = 9.450). In the non-questioning condition, we conducted a one-way ANCOVA with synergy type (non-synergy and synergy) as a between-group factor and product memory as a dependent variable. The results (see Table 6) reveal that product recall between the non-synergistic group (*M*_*non–synergy*_ = 4.316; *t* = 0.000, *p* = 0.996) and the synergistic group (*M*_*synergy*_ = 4.313) did not significantly differ, and product identification between non-synergistic and synergistic groups (*M*_*synergy*_ = 9.375, *M*_*non–synergy*_ = 9.158; *t* = 0.096, *p* = 0.758) also did not differ.

**TABLE 5:** Comparison of non-synergistic stimulation and synergistic stimulation in the questioning condition.

Variable	Media exposure		*t*	Significance
	Non-synergy (B-B and I-I)	Synergy (B-I and I-B)		
Product Recall	4.550	5.833	7.378	0.010
Product Identification	9.450	10.458	4.527	0.039

**TABLE 6:** Comparison of non-synergistic stimulation and synergistic stimulation in the non-questioning condition.

Variable	Media exposure		*t*	Significance
	Non-synergy (B-B and I′-I′)	Synergy (B-I′ and I′-B)		
Product Recall	4.316	4.313	0.000	0.996
Product Identification	9.158	9.375	0.096	0.758

In this study, we verified that the two media with different information processing levels could produce synergistic effects, so H2b is validated. In the non-questioning condition, even though the laptop home page and the laptop forum were different in their form, there was no difference in the level of information processing because of the button in the forum page without automatic pop-up questions. In the questioning condition, the difference between the laptop home page and the laptop web forum came from the difference in the communication method (one-way broadcast and two-way interactive).

This study concludes that the difference between the two stimulations will produce a synergistic effect, which is consistent with previous studies. Previous studies focused on the impact of different media forms on marketing communication. For example, perceived pattern differences between broadcast media and dynamic video media can produce a synergistic effect (Edell and Keller, 1989). The difference in the perceived pattern has the advantage of providing multiple coding channels (Voorveld et al., 2011). Some studies have focused on the differences in information load and information origin (Wang and Nelson, 2006; Chatterjee, 2012) from which the synergistic effect might come. In short, inter-media differences in the cognitive coding process lead to a richer memory structure. In this study, the difference of information processing level between broadcast and interactive media resulted in a synergistic effect in product memory that is consistent with our proposed theory.

## General Discussion

In the present research, we provide strong support across three studies for the thesis that the combination of online broadcast media and interactive media increases the effect of marketing communication on purchase intention and product memory because of the synergistic effect. In Study 1, the joint stimulation of online broadcast media and interactive media led to a synergistic effect on consumer purchase intention (H1). In Study 2, we empirically verified our proposed theory that a synergistic effect occurs only when the interaction between online broadcast media and interactive media actually works (H2a). In Study 3, we demonstrate that, when the information processing level is different, a synergistic effect of online broadcast media and interactive media occurs (H2b).

### Theoretical Implications

This research contributes to the literature streams on marketing synergy, media planning, and integrated marketing communication. First, we extend research on marketing synergy by introducing a new marketing media classification.

Second, we extend research on media planning in communications to reveal that a media portfolio is effective only when the two media interact with each other and a difference in media information processing level between the two media is present. The sequential stimulation from two or more distinct media reinforces the marketing message, leading to a stronger cognitive structure. There is no synergistic effect if there is no interaction and no difference between online broadcast media and interactive media.

Third, previous research on integrated marketing communication has focused on the outcome variables that can measure the effect of integrated marketing communication (Schultz et al., 2012). The present research, however, demonstrates that these outcome variables are affected by product or brand memory.

### Managerial Implications

This research also has an important managerial implication for marketers. First, it has been found that online media synergy between different media can lead to a more positive purchase intention and product memory than repeated media advertisement. In the internet environment, marketers need to integrate different media to bring out communication breadth. Therefore, how to classify media to form a media portfolio becomes an important issue. Based on an online broadcast and interactive classification framework, marketers can build a media portfolio to achieve their marketing goals.

Furthermore, marketers should adapt the multimedia interaction to the characteristics of the marketing media portfolio. In the practice of real marketing, if the time interval between the two media stimulations is too long or the theme difference of the two media stimulations is too great, then the two-media synergistic effect does not work due to the absence of a searching prompt.

Finally, marketers should create an online media portfolio with different information processing levels. In practice, if there is no significant difference in interactive dimensions between the media, such as multiple product web pages, there will be the result of no synergistic effect occurrence and of the multimedia portfolio degenerated into a repetition of web pages.

## Data Availability Statement

The raw data supporting the conclusions of this article will be made available by the authors, without undue reservation.

## Ethics Statement

Ethical review and approval was not required for the study on human participants in accordance with the local legislation and institutional requirements. The patients/participants provided their written informed consent to participate in this study.

## Author Contributions

YG conceived and designed the study. Both authors performed the experiments, wrote the manuscript, reviewed and edited the manuscript, and read and approved the manuscript.

## Conflict of Interest

The authors declare that the research was conducted in the absence of any commercial or financial relationships that could be construed as a potential conflict of interest.

## Publisher’s Note

All claims expressed in this article are solely those of the authors and do not necessarily represent those of their affiliated organizations, or those of the publisher, the editors and the reviewers. Any product that may be evaluated in this article, or claim that may be made by its manufacturer, is not guaranteed or endorsed by the publisher.
