# Supplementary material for: Synergistic Effect Between Online Broadcast Media and Interactive Media on Purchase Intention
Source: Front Psychol. 2021 Dec 23;12:781743. doi: 10.3389/fpsyg.2021.781743 (PMC8733660; doi:10.3389/fpsyg.2021.781743)
Supplement: Supplementary file 1 [file Data_Sheet_1.docx]

Appendix 1 Figure A1

Multimedia

Synergy

Product Recall

Purchase Intention

**Figure A1 Conceptual Model**

Product Identification

Appendix 2 Selected Research Materials

(1) A mobile phone advertising claims are as follows:

| Unique smart touch in internal and external dual-screen, transparent screen in flip, and intelligent control experience. |
| --- |
| High-definition, ultra-smooth CMMB mobile TV function, equipped with an external antenna to ensure signal stability. Multiple programs can be watched at any time. |
| Real-time recording and playback function, 32GB large expansion of memory, can handle multiple tasks while recording a long TV program. |
| When you answer a call, the Dis-80 automatically records the TV program for playback, ensuring that no critical passage or meaningful moment is ever missed. |
| Integration of a full set of mobile applications, including financial stock market, text scanning, sending and receiving e-mail and other functions. |
| Dis-80 supports 720p-quality, high-definition audio and video playback, accompanied by excellent sound quality through the speaker to communicate, anytime, anywhere and to ensure an unprecedented listening experience. |
| Dis-80 also supports RMVB/RM video format, allowing you to enjoy online video resources. The playback process can be controlled both inside and outside the dual screen. Enjoy the dazzling appearance of the large screen. |
| 3G/WLAN (WAPI/WIFI) high-speed Internet access with information breakthrough territory restrictions, allowing you to surf the Internet everywhere in the world. |
| Unique transparent clamshell design, low-resistance touch screen, precise control, to bring you an exquisite experience. |
| CMMB mobile TV with clear and smooth program, more real-time recording and playback so as not to miss any programs. |
| Mobile TV programs can be manipulated in the dual screen; automatic switching between horizontal and vertical screen views, offering a variety of ways to watch video programs, including panoramic views and audition. |
| A full set of intelligent applications by Almighty King, just use the phone to take pictures, you can quickly convert printed text into an electronic document, and automatically load the content of a business card into your address book. |
| Quickoffice can view and edit Word, PPT, Excel, PDF and other file formats, but also support mobile phone and other applications. |
| Dis-80 is equipped with industry-leading smart handwriting input technology that can even recognize cursive writing efficiently and quickly. |
| Supports 720P high-definition video playback and video shock showing. |
| 3G high-speed Internet supporting video calls and other communications across time and space as if they were in person; supports wireless local area network (WAPI/WIFI); enjoy the benefits of technology, speedy access, and unlimited information. |

(2) A laptop advertising claims are as follows:

| Flexible tracking point (Track Point) for more convenient operation. |
| --- |
| Screen with borders, BC border design, double buckle design to better protect the screen, more user friendly top keyboard lights. |
| Beautiful multi-color appearance, hard shell protection, TX washable titanium magnesium alloy, and scratch resistant. |
| Unique seven lines of keyboard, more features, the best keyboard feel, waterproof. |
| Wireless network card antenna embedded in the screen on both sides, in a high position for a better signal. |
| Active keyboard protection system (APS) for better data security. |
| A key recovery function: if the system is damaged, you can restore it with the last backup. |
| Embedded security subsystem from the software and hardware to solve data security issues. |
| Dolby 5.1 surround sound field design, effective noise reduction, Chunmei output sound quality, guaranteeing an immersive experience. |
| Using a new generation of Intel Centrino dual-core processor technology to launch a new sensory revolution. |
| With a convenient pointing point (Track Point), a substantial move pointer and drag operation is simpler. |
| Screen with no border design for a better visual experience, carbon fiber matte texture, and a fashionable thin border. |
| Titanium magnesium alloy external body with a steady style. |
| High-touch keyboard for comfort and convenient long-term operation. |
| More reasonable wireless card, good signal reception, easy to wear, long-term warranty. |
| Active Protection System (APS) automatically protects the hard disk drive against possible data corruption. |
| New key recovery function that supports personalized system recovery without reinstalling the system. |
| Store and run passwords and related information dedicated to data and program protection through the Embedded Security Subsystem. |
| JBL stereo sound system with bright and clear sound and a high degree of noise reduction. |
| A new generation of Intel Centrino dual-core technology supporting the “high-speed, thin, long drive” concept for a better high-speed processing experience. |

Appendix 3 Questionnaire for the study

(1) Purchase intention and product attitude measurement questionnaire

Please answer the following questions about the products featured in the ads you've just seen. Score values range from 1 (completely disagree) to 7 (completely agree); 4 indicates uncertainty. Please tick √ the corresponding score.

1. How likely are you to buy the products shown in the ad in the next 12 months?

A. 99% likelihood of making a purchase

B. 90% likelihood of making a purchase

C. 80% likelihood of making a purchase

D. 70% likelihood of making a purchase

E. 60% likelihood of making a purchase

F. 50% likelihood of making a purchase

G. 40% likelihood of making a purchase

H. 30% likelihood of making a purchase

I. 20% likelihood of making a purchase

J. 10% likelihood of making a purchase

K. 1% likelihood of making a purchase

2. Based on the information given by the media just now, please choose your attitude towards the product in the advertisement.

| 1 | Not good | 1 | 2 | 3 | 4 | 5 | 6 | 7 | Good |
| --- | --- | --- | --- | --- | --- | --- | --- | --- | --- |
| 2 | Dislike | 1 | 2 | 3 | 4 | 5 | 6 | 7 | Like |
| 3 | No desire | 1 | 2 | 3 | 4 | 5 | 6 | 7 | Desire |

(2) Product memory measurement questionnaire

This part of the questionnaire consists of two separate measurements—product recall and product identification.

Part I: Please recall the ads you have just browsed and write down all the information you can remember. Write only one promotional message or product feature in each line.

| 1、 |
| --- |
| 2、 |
| 3、 |
| 4、 |
| 5、 |
| 6、 |
| 7、 |
| 8 |
| 9、 |
| 10、 |
| 11、 |
| 12、 |
| 13、 |
| 14、 |
| 15、 |
| 16、 |

Part II: Based on your memories, please indicate whether the following information was presented in the advertisement you viewed by placing a tick mark (√) on either “yes” or “no”.

| The mobile phone being advertised is Disdara (Dis-80). | Yes | No |
| --- | --- | --- |
| Which phone has a foldable dual screen and smart touch function? | Yes | No |
| The phone’s CMMB mobile TV function is available through the built-in antenna. | Yes | No |
| The phone’s 32G memory supports instant recording. | Yes | No |
| The phone automatically records the TV program while you’re answering a call. | Yes | No |
| The mobile phone’s full set of mobile applications includes business card processing. | Yes | No |
| The phone supports 1080p-quality HD video playback. | Yes | No |
| The phone supports both RMVB and RM video formats. | Yes | No |
| The phone gives access to the Internet via LAN/3G. | Yes | No |
| Is the advertising claimed that “Technology touch mind, Wisdom solve the problem”? | Yes | No |
| The phone features a resistive touch screen. | Yes | No |
| Which mobile phone is CMMB type? | Yes | No |
| The phone can be manipulated through the external screen. | Yes | No |
| Is the mobile phone available in processing business card? | Yes | No |
| The phone supports online games. | Yes | No |
| The phone’s smart input technology can recognize cursive handwriting. | Yes | No |
| The phone supports 720p video playback. | Yes | No |
| The phone supports video chat. | Yes | No |

| The notebook finger bar function can perform drag-and-drop operations. | Yes | No |
| --- | --- | --- |
| The notebook’s frame is made of matte carbon fiber. | Yes | No |
| The notebook’s outer body is made of a titanium magnesium alloy. | Yes | No |
| The notebook features a with seven key keyboard . | Yes | No |
| The notebook’s wireless card has a long warranty. | Yes | No |
| The notebook has an active protection feature to protect the floppy drive. | Yes | No |
| The notebook has a key recovery function to support personalized recovery settings. | Yes | No |
| The notebook has an external security subsystem. | Yes | No |
| The notebook’s sound system is made by JBL. | Yes | No |
| The notebook’s dual core adheres to the original “high-speed, light, smooth” concept. | Yes | No |
| The notebook has a wrapping border. | Yes | No |
| The notebook’s upper keyboard has a double buckle design. | Yes | No |
| The titanium-magnesium alloy of the notebook is processed by TX process. | Yes | No |
| The notebook’s keyboard is waterproof. | Yes | No |
| The notebook’s antenna is embedded in the top of the screen. | Yes | No |
| The notebook has a Dolby 5.1 surround sound field design. | Yes | No |
